# Sigma-1 receptor is involved in diminished ovarian reserve possibly by influencing endoplasmic reticulum stress-mediated granulosa cells apoptosis

**DOI:** 10.18632/aging.103166

**Published:** 2020-05-14

**Authors:** Lile Jiang, Jinquan Cui, Cuilian Zhang, Juanke Xie, Shaodi Zhang, Dongjun Fu, Wei Duo

**Affiliations:** 1 Reproductive Medical Center, People's Hospital of Zhengzhou University, Zhengzhou, Henan, China; 2 Department of Obstetrics and Gynecology, The Second Affiliated Hospital of Zhengzhou University, Zhengzhou, Henan, China; 3 School of Pharmaceutical Sciences and Collaborative Innovation Center of New Drug Research and Safety Evaluation, Zhengzhou University, Zhengzhou, Henan, China

**Keywords:** sigma-1 receptor, diminished ovarian reserve, endoplasmic reticulum stress, granulosa cells, apoptosis

## Abstract

Sigma non-opioid intracellular receptor 1 (sigma-1 receptor), a non-opioid transmembrane protein, is located on cellular mitochondrial membranes and endoplasmic reticulum. Current research has demonstrated that sigma-1 receptor is related to human degenerative diseases. This study is focused on the effects of sigma-1 receptor on the pathophysiological process of diminished ovarian reserve (DOR) and granulosa cells (GCs) apoptosis. Sigma-1 receptor concentration in follicular fluid (FF) and serum were negatively correlated with basal follicle-stimulating hormone (FSH) and positively correlated with anti-mullerian hormone (AMH), antral follicle count (AFC). Sigma-1 receptor reduction in GCs was accompanied by endoplasmic reticulum stress (ERS)-mediated apoptosis in women with DOR. Plasmid transfection was used to establish SIGMAR1-overexpressed and SIGMAR1-knockdown human granulosa-like tumor (KGN) cell and thapsigargin (TG) was used to induce ERS KGN cells. We found that KGN cells treated with endogenous sigma-1 receptor ligand dehydroepiandrosterone (DHEA) and sigma-1 receptor agonist PRE-084 showed similar biological effects to SIGMAR1-overexpressed KGN cells and opposite effects to SIGMAR1-knockdown KGN cells. DHEA may improve DOR patients' pregnancy outcomes by upregulating sigma-1 receptor and downregulating ERS-mediated apoptotic genes in GCs. Thus, sigma-1 receptor may be a potential ovarian reserve biomarker, and ligand-mediated sigma-1 receptor activation could be a future approach for DOR therapy.

## INTRODUCTION

Infertility is a common disease and affects more than 15% of reproductive age couples [1]. Diminished ovarian reserve (DOR) is found in approximately 10% of infertile women [2, 3]. Ovarian reserve means the number and quality of oocytes that are produced by ovaries both in the follicular phase of the natural menstrual cycle and following injection of follicle stimulating hormone (FSH) in the assisted reproductive technology (ART) protocol [2, 4, 5]. Patients with DOR are always associated with increased miscarriage rates and aneuploidy rates [6, 7]. Currently the cause of DOR is unknown, and there is a lack of an efficient treatment in the present standard fertility protocol [8–10]. Many research groups have focused on the transcriptomes between oocytes and ovarian granulosa cells (GCs) [11–14]. They have reported that follicular atresia during the progression of DOR is primarily induced by apoptosis of GCs, so our research focus on GCs apoptosis.

Sigma-1 receptor, a non-opioid transmembrane protein, is mostly located on mitochondrial membranes and the endoplasmic reticulum (ER), and is expressed in a broad range of tissues [15–18]. It has also been reported the it could be detected in extracellular fluid [19]. As a molecular chaperone, sigma-1 receptor has multiple cellular functions and is involved in a series of diseases [20–22]. Studies have confirmed that sigma-1 receptor is an endoplasmic reticulum stress (ERS)-associated membrane chaperone protein [23–25]. Recent studies have demonstrated that sigma-1 receptor is a degenerative disease-related protein via protein-protein interactions. Stimulation of sigma-1 receptor showed a neuroprotective effects in Parkinson’s disease, Alzheimer’s disease, amyotrophic lateral sclerosis, and stroke [26–32]. Vilner et al. first reported that sigma-1 receptor is highly expressed in several types of cancer cells at the proliferation stage [33]. Some *in vivo* and *in vitro* studies indicated that sigma-1 receptor induced by ligands or over-expression of the sigma-1 receptor protects cell against apoptosis [31, 32, 34, 35]. Compared to other organs, the ovary is very sensitive to aging [36, 37]. We hypothesized that sigma-1 receptor may participate in the pathological process of DOR. The first part of our study investigated the expression of sigma-1 receptor in the follicular fluid (FF), serum and GCs of women with DOR. Considering that GCs apoptosis plays a major role in the progression of DOR, we also examined several ERS-mediated apoptosis target genes to explore the possible molecular mechanisms. In 2000, Casson et al. [38] first reported that dehydroepiandrosterone (DHEA) supplementation has a series of beneficial effects on ovarian function in patients with DOR; other studies [39, 40] have confirmed its validity. Numerous studies have demonstrated that DHEA is an endogenous ligand of sigma-1 receptor [41–44]. In the latter part, human granulosa-like tumor (KGN) cells were chosen as the study object. We established SIGMAR1-overexpressed and SIGMAR1-knockdown and ERS KGN cell lines, and aimed to verify if DHEA play a similar role like sigma-1 receptor agonist PRE-084 in ovarian GCs’ apoptosis by sigma-1 receptor activation.

## RESULTS

### Sigma-1 receptor expression in the ovaries of women of childbearing age

**Figure 1:**
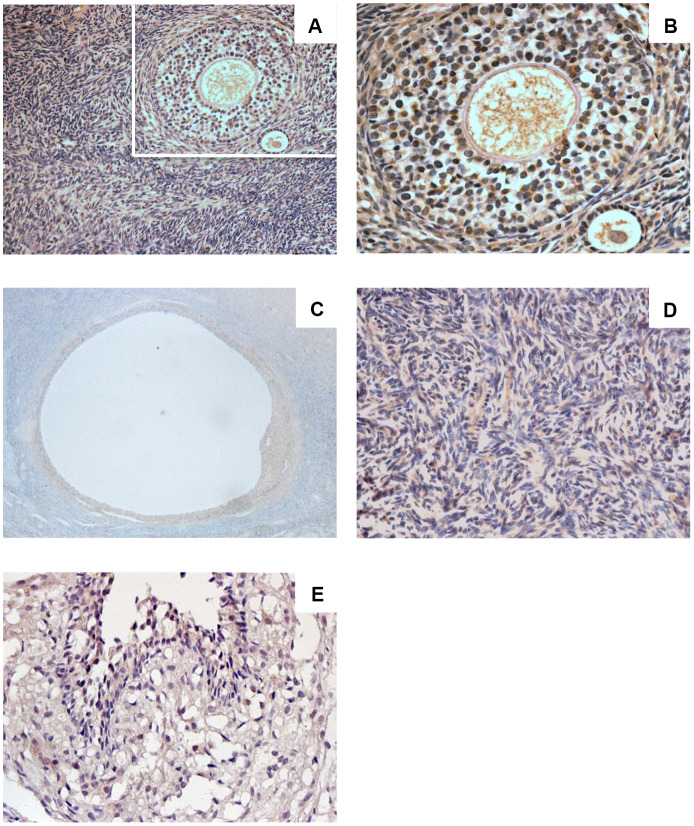
**Sigma-1 Receptor Expression in Ovary Tissue.** Ovarian cortex in women of childbearing age (**A**, ×200) (**C**, ×400) showed intense immunostaining. Intense immunostaining was observed inovarian granulosa cells and theca cells of growing follicle (**B**, ×400). Granulosa cells of mature follicle also showed intense staining (**D**, ×200). Low immunostaining was observed in human ovarian stromal cells (**E**, ×400).

Sigma-1 receptor was expressed in human ovarian tissue (Figure 1A–1E). Ovarian cortex showed intense immunostaining (Figure 1A, 1B). Intense immunostaining was observed in ovarian granulosa cells and theca cells of growing follicle (Figure 1A, 1C). Granulosa cells of mature follicle also showed intense staining (Figure 1D). Positive immunostaining was observed in the cytoplasm, in luteinized GCs there was very intense staining. Sigma-1 receptor also appeared in ovarian stroma cells with low immune staining (Figure 1E), the results are consistent with the Protein-Atlas database results.

### Clinical characteristics of women with DOR

Oocytes and GCs were obtained from 130 women (46 women with DOR and 84 women with normal ovarian reserve (NOR) undergoing in-vitro fertilization (IVF) cycles. Patients’ clinical characteristics are shown in Supplementary Table 1. In comparison with women with NOR, patients with DOR had higher mean maternal age, duration of infertility, number of IVF cycles, basal FSH levels, and initiating dosage of gonadotropin (*P*<0.05). In addition, these patients had significantly lower serum anti-mullerian hormone (AMH) levels, antral follicular count (AFC), retrieved oocytes, and available embryos (*P*<0.05) (Supplementary Table 1).

### Sigma-1 receptor protein level was decreased in the serum, FF, and GCs of women with DOR

**Figure 2:**
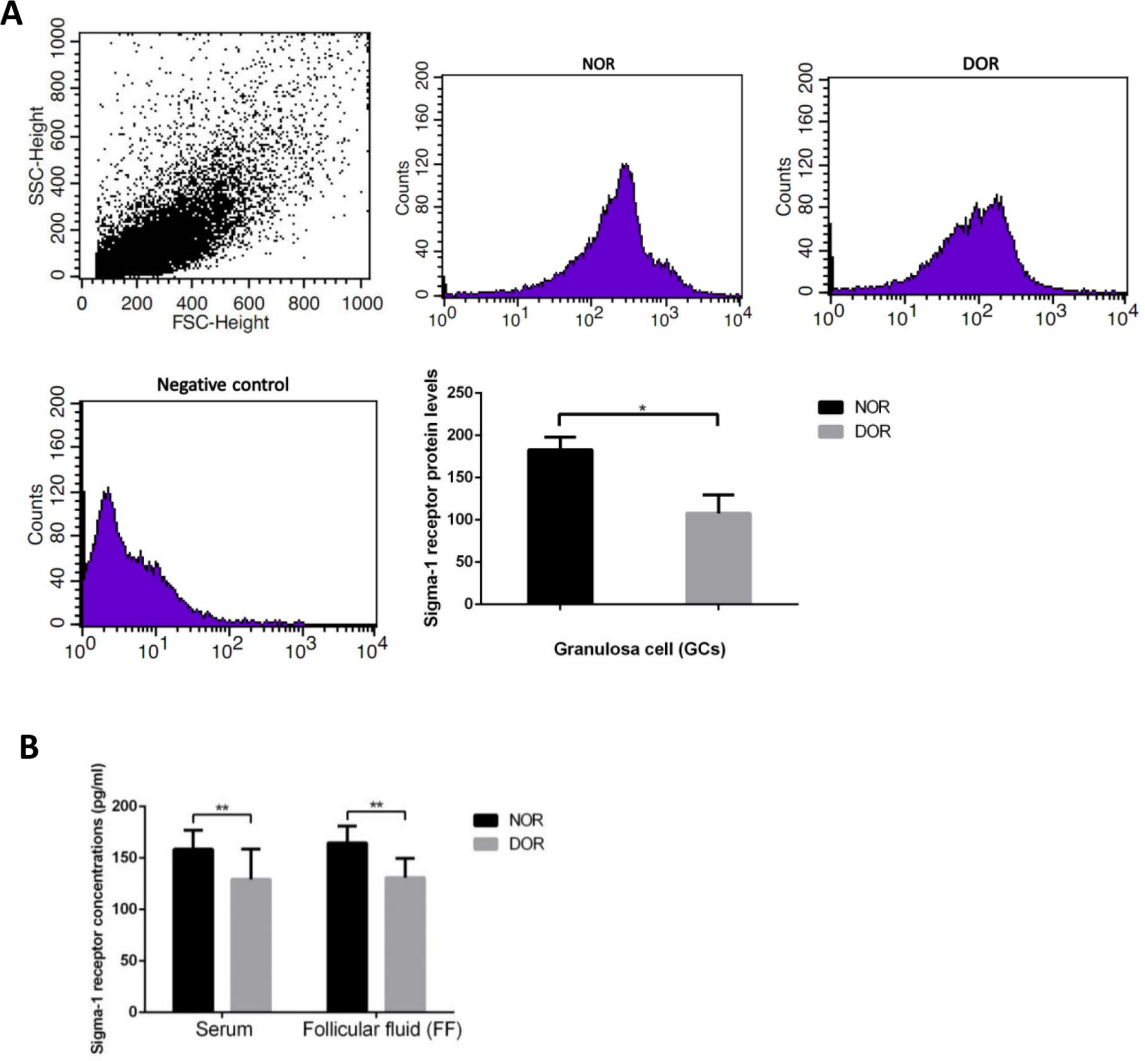
**Sigma-1 Receptor Protein Expression in GCs, serum, and FF from patients with DOR.** (**A**) FCM sorting of sigma-1 receptor protein in patient GCs, low sigma-1 receptor protein level in DOR patients’ GCs. (**B**) Low sigma-1 receptor level in DOR patients’ serum and FF. ** P<0.01.

The result of flow cytometry (FCM) showed that DOR patients’ GCs had much lower sigma-1 receptor protein levels than women with NOR (*P* = 0.031) (Supplementary Table 2, Figure 2A). Serum and FF sigma-1 receptor protein levels of patients are listed in Supplementary Table 3. Sigma-1 receptor concentrations in serum and FF of women with DOR were much lower than in those with NOR (*P*<0.01) (Figure 2B). There were negative correlations of sigma-1 receptor concentration in FF and serum with basal FSH and maternal age (all *P*<0.01) (Table 1). Sigma-1 receptor levels in FF and serum were positively correlated with serum AMH, AFC, retrieved oocytes, and available embryos (all *P*<0.01) (Table 1).

**Table 1:** Significant correlations exist between sigma-1 receptor concentration in FF and serum and ovarian reserve markers (n=130).

	**bFSH**	**AMH**	**AGE**	**AFC**	**No. of oocyte retrieved**	**No. of available embryos**
The level of serum sigma-1 receptor	R = -0.664	R = 0.590	R = -0.556	R = 0.546	R = 0.529	R = 0.536
	P = 0.000**	P = 0.000**	P = 0.000**	P = 0.000**	P = 0.000**	P = 0.000**
The level of FF sigma-1 receptor	R = -0.685	R = 0.682	R = -0.550	R = 0.531	R = 0.609	R = 0.604
	P = 0.000**	P = 0.000**	P = 0.000**	P = 0.000**	P = 0.000**	P = 0.000**

**<0.01.

### The increase of apoptosis rate in the GCs of patients with DOR was accompanied by the increase of ERS-mediated and apoptosis-related gene expression

**Figure 3:**
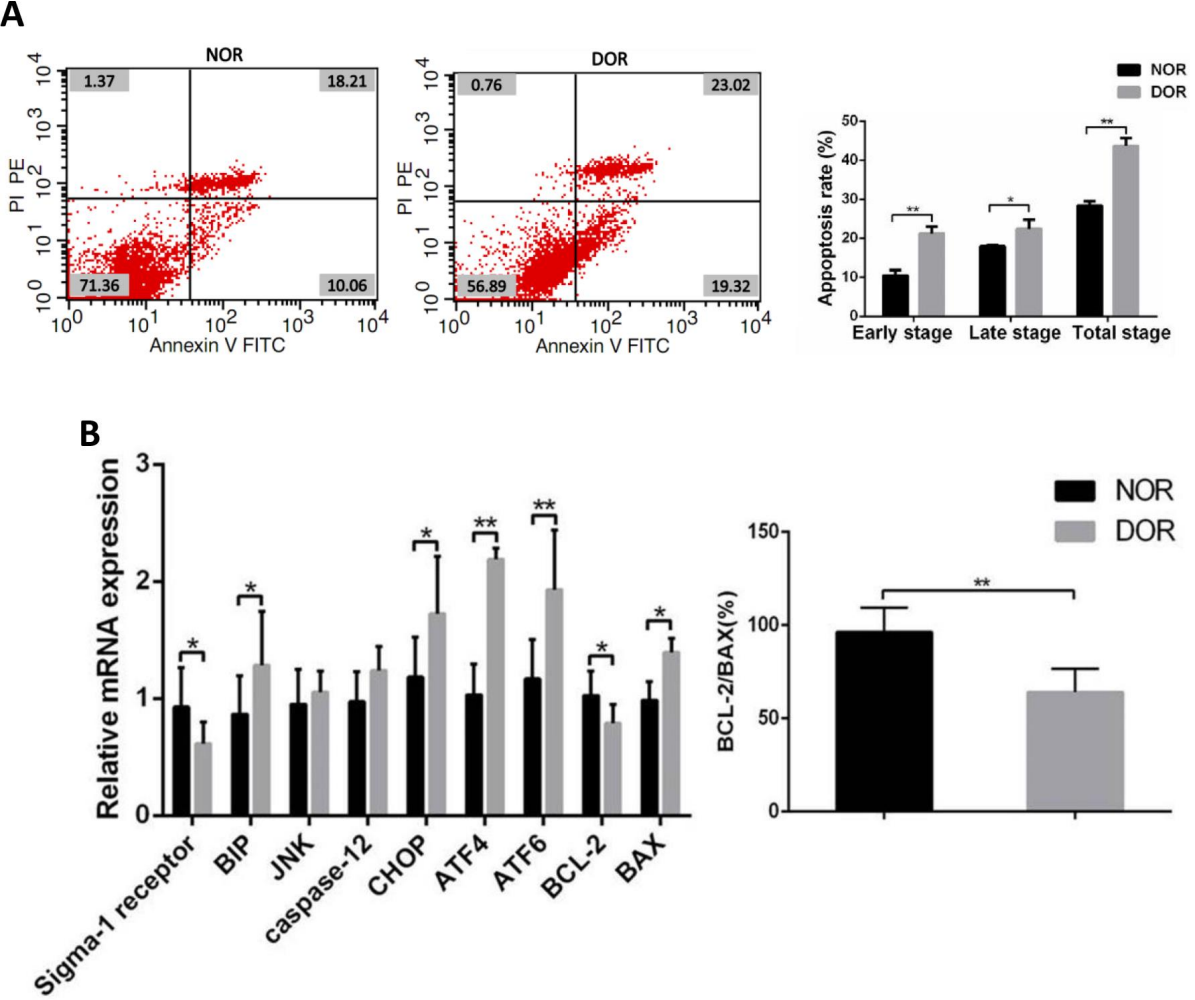
**Apoptotic rate and ERS, mRNA levels of apoptosis-related genes in GCs from patients with DOR.** (**A**) Apoptosis index in patient GCs. (**B**) The mRNA expression of ERS and apoptosis-related genes was increased in patient GCs. *P<0.05; **P<0.01.

FCM showed that in GCs the early apoptosis rate, late apoptosis rate, and total apoptosis rate in patients with DOR were significantly higher than those in women with NOR, respectively (all *P* < 0.05) (Supplementary Table 4 and Figure 3A). Real time quantitative polymerase chain reaction (qRT-PCR) assays showed that mRNA levels of the ERS-mediated genes BIP, CHOP, ATF4, ATF6 and proapoptosis gene BAX were increased in the GCs of women with DOR (all *P*<0.05), and the expression of sigma-1 receptor and apoptosis-related BCL-2, ratio of BCL-2/BAX were decreased (all *P*<0.05). However, JNK, caspase 12 in GCs did not show any statistical differences between the 2 groups (*P*>0.05) (Supplementary Table 5 and Figure 3B).

### DHEA supplementation improved clinical outcomes for patients with DOR

**Figure 4:**
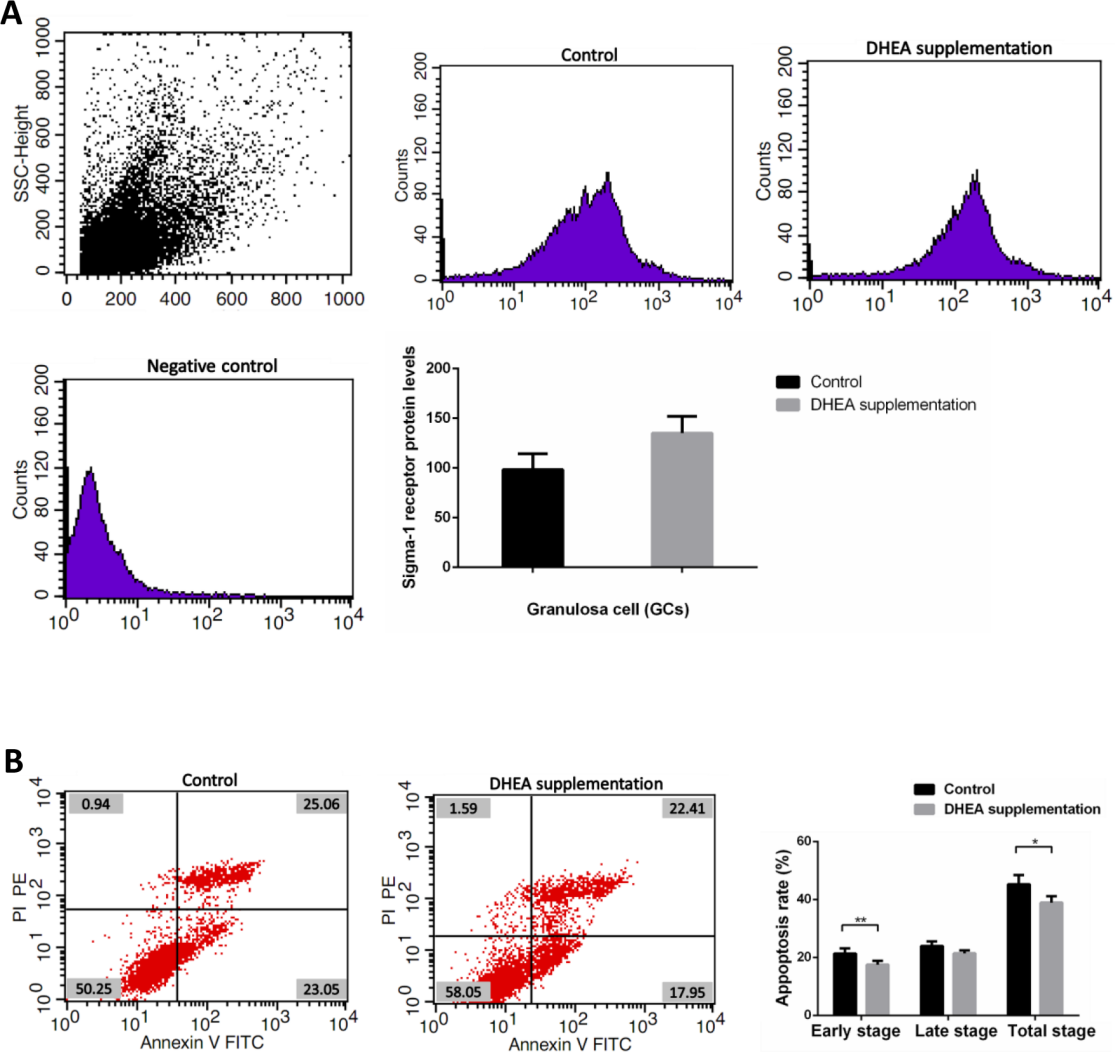
**Sigma-1 Receptor Protein Expression and apoptosis rates in GCs from DOR patients with DHEA supplementation.** (**A**) The sigma-1 receptor protein levels in DHEA supplementation patient GCs. (**B**) Apoptosis index in DHEA supplementation patient GCs.

Clinical data obtained from 89 patients with DOR (31 women with DHEA supplementation and 58 women with control treatment) are shown in Supplementary Table 6. No significant differences appeared between the two groups, including maternal age, duration of infertility, IVF cycles, infertility type (primary or secondary infertility), BMI, AFCs, basal FSH and LH levels, serum AMH level, the use of gonadotrophin, and endometrial thickness. Administration of DHEA increased patients’ available embryos and decreased cancelation rates of IVF cycles (*P*<0.05). Retrieved oocytes, available embryos, embryos transferred, the rate of high quality embryos, clinical pregnancy, abortion, and ectopic pregnancy showed no statistical differences between the two groups (*P* > 0.05) (Supplementary Table 6). Compared with control treatment patients, there was a slight increase of GCs’ sigma-1 receptor protein levels in DOR patients with DHEA supplementation, but no statistical difference between two groups (*P* = 0.052) (Supplementary Table 7 and Figure 4A). FCM showed that GCs’ early apoptosis rate, and total apoptosis rate in DOR patients with DHEA supplementation were significantly lower than control group, respectively (all *P* < 0.05) (Supplementary Table 8 and Figure 4B).

### KGN cells treated with DHEA and PRE-084 showed similar biological effects to KGN cells overexpressing SIGMAR1 and opposite effects to SIGMAR1-knockdown KGN cells

**Figure 5:**
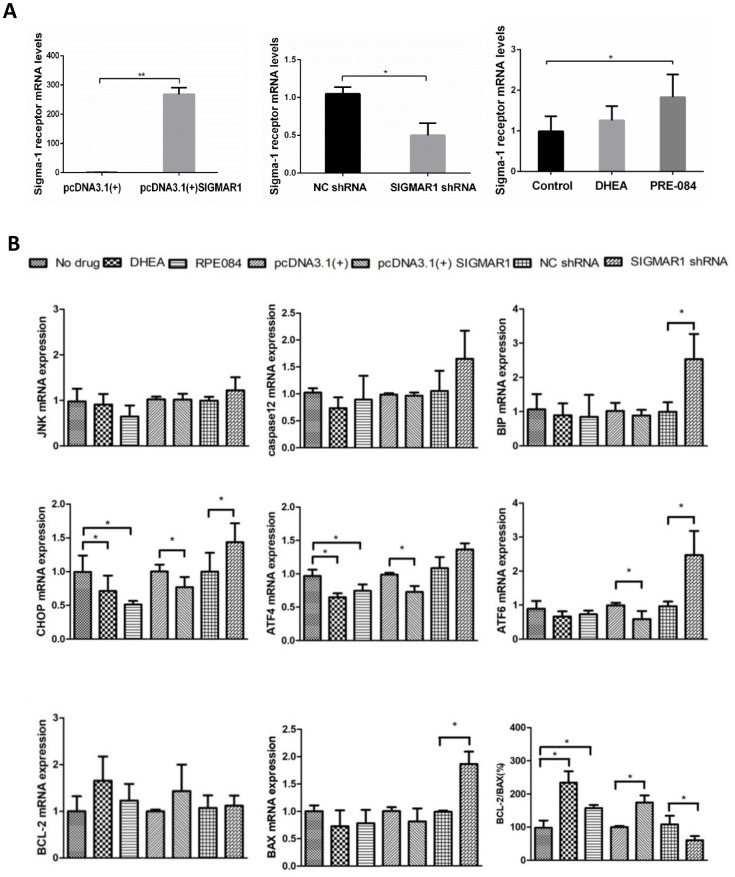
**KGN cells treated with DHEA- and sigma-1 receptor ligands revealed similar biological effects to SIGMAR1-overexpressed cells and opposite effects to SIGMAR1-knockdown KGN cells.** (**A**) Sigma-1 receptor mRNA levels in differently treated KGN cells. SIGMAR1-overexpressed and SIGMAR1-knockdown KGN cells were constructed successfully. (**B**) The mRNA levels of ERS and apoptosis-related genes in differently treated KGN cells. *P<0.05.

The mRNA level of sigma-1 receptor was significantly overexpressed in pcDNA3.1(+)-SIGMAR1 KGN cells, compared to control cells (*P* = 0.000). Sigma-1 receptor mRNA expression was significantly inhibited by SIGMAR1 shRNA KGN cells (*P* = 0.014) (Supplementary Table 9 and Figure 5A). Compared to the control group, KGN cells treated with DHEA and classical sigma-1 receptor agonist PRE-084 had much higher levels of sigma-1 receptor, but only the difference in PRE-084-treated cells was significant (*P*<0.05) (Supplementary Table 9 and Figure 5A). Then, we detected the expression levels of 8 apoptosis-related and ERS-mediated genes by qRT-PCR, including JNK, caspase12, BIP, ATF4, ATF6, CHOP, BCL-2, and BAX. Compared with control cells, ATF4 and CHOP levels were decreased and the BCL-2/BAX ratio was increased in DHEA- and PRE-084-treated KGN cells (*P*<0.05). In SIGMAR1-overexpressed KGN cells, ATF4, ATF6 and CHOP were decreased while the BCL-2/BAX ratio was increased (*P*<0.05). We found that BIP, ATF6, CHOP, and BAX were increased while the BCL-2/BAX ratio was decreased in SIGMAR1-knockdown KGN cells. With regard to JNK and caspase12 mRNA levels there was no significant statistical difference among differently treated KGN cells. (*P*<0.05) (Supplementary Table 9 and Figure 5B).

### The decrease of apoptosis rates in thapsigargin(TG)-induced ERS KGN cells treated with PRE-084/DHEA was accompanied by the decrease of ERS-mediated and apoptosis-related gene expression

Considering of MTT assay and qRT-PCR results, an increase of TG concentration was accompanied by a decrease in KGN cell viability. Compared to the control group, KGN cells treated with 0.5μM TG had lower cell viability (*P*<0.05), KGN cells treated with equal to or greater than 1μM TG had much lower cell viability (*P*<0.01) (Supplementary Table 10). qRT-PCR assays showed that ERS-mediated gene CHOP, BIP and apoptosis-related gene BCL-2, BAX expression levels had no significant difference between 0.5μM TG-induced KGN cells and control group. KGN cells treated with equal to or greater than 1μM had much higher CHOP, BIP mRNA levels and lower BCL-2 mRNA levels, BCL-2/BAX ratios (Supplementary Table 11). We choose optimal concentration of 1μM TG for the subsequent experiments. Flow cytometry showed that early stage apoptotic rate and total apoptotic rate were increased significantly in KGN cells treated with TG (*P*<0.05). qRT-PCR assays showed that mRNA levels of the ERS-mediated genes BIP, CHOP, JNK, caspase12, ATF4, ATF6 were increased in KGN cells treated with TG (all *P*<0.05). Compared with TG-induced KGN cells, both early stage and total apoptotic rates were decreased significantly in TG-PRE-084-treated and TG-DHEA-treated KGN cells (*P*<0.05) (Supplementary Table 12 and Figure 6A), sigma-1 receptor protein levels in TG-PRE-084/DHEA-treated KGN cells were increased (*P*<0.05) (Supplementary Table 13 and Figure 6B), and the expression of apoptosis-related BCL-2/BAX ratios were increased while ERS-mediated genes BIP, CHOP, ATF4, ATF6 mRNA levels were decreased in TG-PRE-084/DHEA-treated KGN cells, BCL-2 mRNA level was increased in TG-PRE-084-treated KGN cells (all *P*<0.05) (Supplementary Table 14 and Figure 6C).

**Figure 6A-B:**
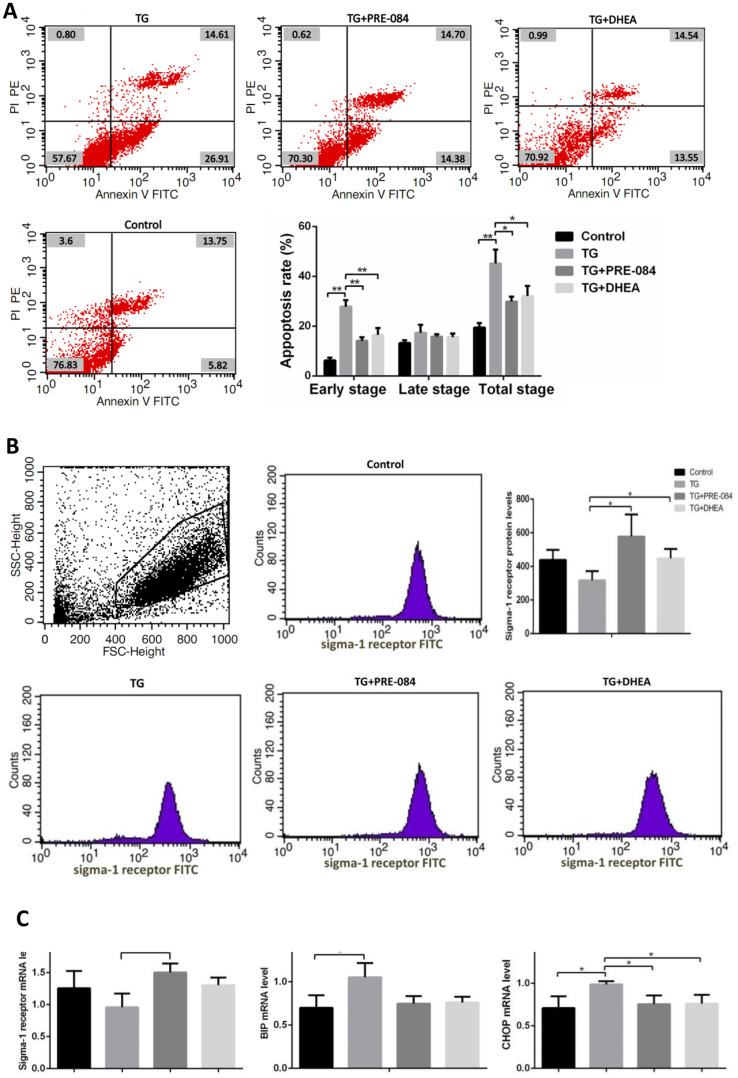
**The decrease of apoptosis rates in thapsigargin(TG)-induced ERS KGN cells treated with PRE-084/DHEA was accompanied by the decrease of ERS-related and apoptosis-related gene expression.** (**A**) Apoptosis index in KGN cells treated with different drugs. (**B**) Flow cytometry sorting of sigma-1 receptor protein in KGN cells treated with different drugs.

**Figure 6C:**
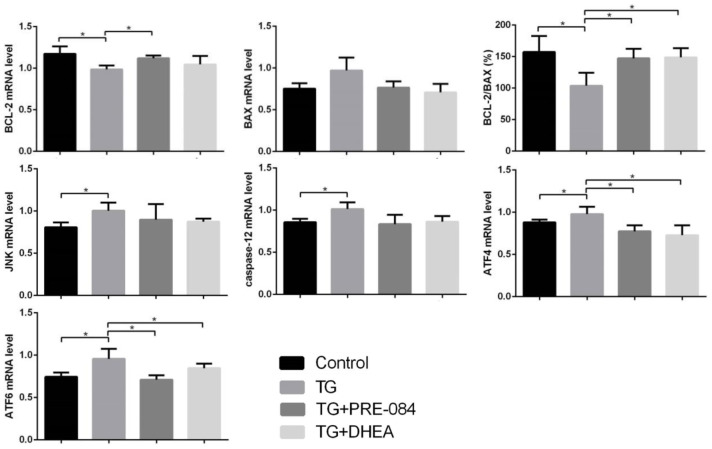
**The decrease of apoptosis rates in thapsigargin(TG)-induced ERS KGN cells treated with PRE-084/DHEA was accompanied by the decrease of ERS-related and apoptosis-related gene expression.** (**C**) The mRNA expression of ERS and apoptosis-related genes in KGN cells treated with different drugs. *P<0.05.

## DISCUSSION

### Sigma-1 receptor expression and ovarian aging

Sigma-1 receptor is a ligand-operated transmembrane chaperone protein that plays a definite neuroprotective effect in several neural degenerative diseases [26–32], and there are reports in the literature that sigma-1 receptor activation promotes nerve cell differentiation in the brain. To some extent, sigma-1 receptor was considered to be an anti-aging and anti-apoptotic protein [45, 46]. The ovary is an organ sensitive to senescence, and its reserve is closely related to age [36, 37]. As far as we know, the connection between sigma-1 receptor and reproductive system diseases has not been reported. The current study investigated sigma-1 receptor expression levels in women’s ovaries, FF, serum, and GCs. We found that sigma-1 receptor was widely expressed in human ovarian tissue. The protein expression levels in FF, serum, and GCs of patients with DOR were decreased. Our results suggested that sigma-1 receptor expression might be associated with ovarian aging.

### Sigma-1 receptor might be a complement to traditional ovarian reserve biomarkers

Patients with DOR present a challenge in the reproductive medical field with poor reproductive treatment outcomes [47]. Ovarian reserve biomarkers are always considered vital markers of pregnancy outcome. Evaluating ovarian reserve is a key step in the ART process [48], serum levels of FSH in the early follicular phase remain the most useful parameter in clinical practice [49]. The majority of studies found that serum AMH level reflects the primordial follicle pool, and it seems to be the most valuable biochemical marker for predicting diminished ovarian reserve in the early stages [50–53]. In this study, we found that the sigma-1 receptor level of FF and serum were negatively correlated with basal FSH and positively correlated with AMH. Patients with low sigma-1 receptor levels in FF and serum were always associated with ovarian reserve dysfunction. From our results, we concluded that sigma-1 receptor is possibly a potential predictor of ovarian function and pregnancy outcome. The application of sigma-1 receptor ligand could be a new research direction in DOR study.

### Sigma-1 receptor is involved in GCs apoptosis and ERS-mediated CHOP apoptosis pathway

In our study, decreased sigma-1 receptor expression is accompanied by increased apoptosis rate in the GCs of women with DOR. Other previous research has found that GCs apoptosis during follicular atresia affects the follicular microenvironment and leads to oocyte apoptosis [54]. Until now, apoptotic intracellular signaling pathways of GCs remained to be determined [48, 49, 55–58]. Earlier research found that GCs apoptosis was predominantly mediated by the cell-death ligand/receptor-dependent pathway [59]. Recent studies have paid more attention to ERS-mediated apoptotic pathways [60–63]. The ER is an important subcellular compartment involved in mitochondrion-dependent apoptosis [64, 65], and ERS is a complicated adaptive reaction caused by certain stimulus [65]. When ERS is too excessive, ER homeostasis fails, then cell apoptotic response occurs [66]. The sigma-1 receptor is a ligand-regulated membrane chaperone protein associated with ERS [67]. Overexpressed sigma-1 receptor counteracts the ERS response and regulates cell survival [68]. The protein BIP is a major chaperone protein [69, 70]. Normally, sigma-1 receptor combined with BIP and forming a complex at the mitochondrial-associated membrane (MAM). During the ERS process, sigma-1 receptor dissociates from BIP, consequently activates BIP function. ERS always activates three branches: protein kinase R-like ER kinase (PERK) pathway, the activating transcription factor 6 (ATF6) pathway, and the inositol-requiring enzyme 1 (IRE1) pathway [71, 72]. Following extensive ERS, cell apoptosis is initiated by transcription factor CCAAT/enhancer-binding protein (C/EBP)-homologous protein (CHOP), caspase 12, or the c-Jun NH 2-terminal kinase (JNK)-dependent pathway activation [71, 73–76]. The three apoptotic pathway functions overlap in many cell types [71, 76]. Lin et al. reported that apoptosis is increased in cultured GCs of goat ovaries through the ERS-mediated CHOP pathway [60]. Meunier and Hayashi found that under cellular stress conditions, sigma-1 receptor ligands increase the expression of the anti-apoptotic gene BCL-2 [77]. Our results showed that decreased sigma-1 receptor expression was accompanied by an increase in the expression levels of apoptosis-related BAX and ERS-mediated BIP in the GCs of patients with DOR. By detecting mRNA levels of ERS-mediated apoptosis pathway downstream molecules, we found that JNK and caspase12 were more highly expressed in the GCs of DOR patients, but the differences were not statistically significant. In addition, mRNA levels of ATF4, ATF6, and CHOP were significantly higher than in the GCs of women with DOR. Therefore, we believe that sigma-1 receptor activation is mainly involved in the ERS-mediated ATF4/ATF6-CHOP apoptosis pathway during the progression of DOR.

### Supplementation with DHEA may improve the pregnancy outcome of patients with DOR via up-regulation of sigma-1 receptor

In the last several decades, several studies explored the relationship between DHEA and neurodegenerative diseases [78–80]. It was found that age-dependent DHEA reduction can cause a series of age-related degenerative diseases, including Alzheimer’s disease and Parkinson disease [81, 82], and DHEA as a sigma-1 receptor ligand became a therapeutic candidate in degenerative diseases [44, 83]. DHEA is the first intervention used in the treatment of DOR [55]. Nowadays, DHEA is routinely applied in DOR clinical practice [39, 40]. Several publications have confirmed that DHEA supplementation can improve the ovarian response of gonadotropin stimulation and increases oocyte yield, embryo number, and clinical pregnancy rate [10, 84–87]. Our current study also confirmed the drug's benefit. Some studies have found that DHEA supplementation seems to improve DOR patients’ ovarian reserve, and appears to function by acting on the androgen receptors that are expressed on the granulosa cells and ovarian stroma [9, 88]. Considering that DHEA has multi-biological functions, our work tried to explain its possible mechanism from a non-specific sigma-1 receptor ligand aspect. After more than 2 months regular DHEA supplementation, the sigma-1 receptor protein of DOR patients’ GCs had a slight increase. KGN cells treated with DHEA revealed similar biological effects to KGN cells treated with classic sigma-1 receptor agonist PRE-084 and KGN cells overexpressing SIGMAR1, while opposite effects were observed in SIGMAR1 knockdown KGN cells. We also found that DHEA supplementation could improve TG-treated KGN cells’ sigma-1 receptor protein level. This indirectly demonstrated that the receptor and ligand interactions between sigma-1 receptor and DHEA, and DHEA might affect GCs apoptosis and ERS by upregulating sigma-1 receptor expression level.

### Sigma-1 receptor ligands might decrease GCs apoptosis by the ERS-CHOP pathway

TG is a classic ERS inducer, which could activate CHOP, caspase 12 and JNK three cell apoptosis pathway, as shown in TG-treated KGN cells’ qRT-PCR results. Our results showed traditional sigma-1 receptor agonist PRE-084 and DHEA showed anti-apoptotic actions in TG-treated KGN cells through downregulation of ERS-mediated gene. Since sigma-1 receptor does not have traditional receptor properties, defined receptor antagonist or agonist activity could always be inaccurate [23]. Sigma-1 receptor activation by ligands may lead to protein conformational changes, leading to the dissociation of sigma-1 receptor from BIP and affecting the biological action of counterpart proteins. We detected that sigma-1 receptor activation causes the ERS key protein, CHOP, to decrease and influences the balance between anti-apoptotic gene BCL-2 and pro-apoptotic gene BAX. This could cause modulation of cell proliferation and apoptosis.

### The decrease of apoptosis rates was accompanied by the increase of sigma-1 receptor protein levels

After more than 2 months regular DHEA supplementation, the increase of sigma-1 receptor protein level in DOR patients’ GCs was accompanied by decrease of apoptosis rates. Our study revealed that sigma-1 receptor ligands and plasmid administration affected the expression of BIP, CHOP, ATF4, and ATF6, which suggested that sigma-1 receptor activation is closely related to the CHOP apoptotic pathway. Sigma-1 ligand could be a new treatment to prevent the DOR process.

To summarize briefly, sigma-1 receptor may be a potential ovarian reserve biomarker. DHEA may improve DOR patients' pregnancy outcomes by upregulating sigma-1 receptor and downregulating ERS-mediated apoptotic genes in GCs. We believe that sigma-1 receptor ligand-based therapies will require us to further investigate the molecular mechanisms that govern sigma-1 receptor’s regulation in cellular stress and homeostasis. Thus ligand-mediated sigma-1 receptor activation could be a future approach for DOR therapy. Downstream molecules targeted by sigma-1 receptor will be our future focus.

## MATERIALS AND METHODS

### Eligibility criteria

### Immunohistochemistry part eligibility criteria

Exclusion criteria were chromosomal abnormalities, autoimmune diseases, ovarian surgery, history of cancer, polycystic ovary syndrome (PCOS), or chemoradiotherapy.

### Patient eligibility criteria

Patients receiving first IVF cycles between May 2016 and October 2016 in the Reproductive Medical Center of the People’s Hospital of Zhengzhou University were enrolled in the study, without excluding patients whose infertility was caused by male factors. The following situations led to exclusion (patients with NOR): (1) PCOS, as diagnosed by the Rotterdam criteria [89]; (2) ovarian oophorectomy; (3) ovarian cystectomy; (4) receiving traditional Chinese medicine treatment or acupuncture with adjuvant therapy; (5) receiving cytotoxic drugs or pelvic irradiation for malignancy; (6) aged more than 38 years; (7) serum AMH below 2.0 ng/mL; (8) basal serum FSH > 10 mIU/mL on Day 2-4 of the menstrual cycle; (9) low basal AFC < 6 on Day 2-4 of the menstrual cycle; (10) DHEA supplementation during the ART process. Patients with DOR had to meet the above exclusion criteria 1-5, 10 and inclusion criteria: basal serum FSH > 10 mIU/mL or serum AMH < 1.17ng/ml. To control the heterogeneity, only patients received gonadotropin-releasing hormone antagonist (GnRH-ant) ovarian stimulation protocol could be included.

### DHEA supplementation part eligibility criteria

Patients underwent oocyte retrieval at the reproductive center in the People’s Hospital of Zhengzhou University between December 2017 and May 2018 were enrolled in the study. The inclusion and exclusion criteria for patients with DOR were as line 254 described.

### Immunohistochemistry

Human ovarian histological sections were obtained from archived wax blocks in the department of pathology in the affiliated second hospital of Zhengzhou. According immunohistochemistry part eligibility criteria, only 9 archived ovarian wax blocks were included. Routine immunohistochemical staining techniques were used in human ovary sections (primary antibody: anti-OPRS1 antibody (Abcam, USA, ab89655, 1:200, incubated 12 hours, 4°C). A streptavidin–biotin (SP) immunohistochemistry kit (Solarbio, China; SP0041) was used to visualize the antibody binding to the tissues. Negative control sections were incubated with PBS instead of the primary antibody. All slides were observed with a digital microscope ECLIPSE TS100 (Nikon, Japan). Positive reaction was nucleus or cytoplasm appeared to be brown.

### Clinical characteristics collection

According patient eligibility criteria on line 245, there were 130 women (46 women with DOR and 84 women with NOR) could be included. Each patients’ general clinical characteristics and oocyte retrieval cycle were recorded.

In DHEA supplementation part, in total 89 patients (31 women in DHEA supplementation group and 58 women in control group). All patients’ general clinical characteristics and first oocyte retrieval cycle were recorded. All patients were followed up for more than 7 months after embryo transfer.

### Collection of serum, FF, and ovarian GCs

Serum was collected in vacutainer with yellow cap from patients at their 3^rd^ day of menstrual cycle, then immediately separated by centrifugation (10 min, ×3500/rpm) All patients underwent controlled ovarian hyperstimulation with GnRH-ant protocol as usually done in our center. After oocyte retrieval, the first aspirated follicle was used for enzyme-linked immunosorbent assay (ELISA) testing. The residual FF was collected for separation of GCs with lymphocyte separation solution (TBD, LTS1077, China) (10 min, ×1800/rpm). The first aspirated follicle was also immediately separated by centrifugation (10 min, ×1500/rpm). Patients’ serum and FF sigma-1 receptor protein concentrations were measured by ELISA kit (Mlbio, ml01637941, China, 1:5 dilution).

### Flow cytometry

Determination of cells’ protein level: After cell fixation and penetration, cells were incubated with the OPRS1 Antibody (N-term) (Abgent, AP2747A-400, China, 1:25 dilution) and Fluorescein (FITC)-conjugated affinipure goat anti-rabbit IgG(H + L) (Jackson Immunoresearch, USA, 1:25 dilution). Apoptotic cells were detected by FITC annexin V apoptosis detection kit I (BD pharmingen, 556547, USA). Then cells were assessed by FACSCalibur (BD Biosciences, USA).

### Construction of SIGMAR1 knockdown or overexpressing KGN cell lines

SIGMAR1 short hairpin RNA pGPU6/GFP/Neo-shNC plasmid and SIGMAR1 pcDNA3.1(+) plasmid were constructed by GenePharma (China). Empty vectors were used as negative control. Transfection was performed using the manufacturer's protocols for Lipofectamine® 2000 (Invitrogen, Carlsbad, CA). The efficiency of knockdown or overexpression was assessed at the mRNA level by qRT-PCR.

### Construction of TG-induced ERS KGN cell lines

The KGN cell line was kindly provided by Professor Mu Y (Chinese PLA general hospital, China), and its biological characteristics have been described in the reference [90]. KGN cells were maintained in Dulbecco's Modified Eagle's Medium/Nutrient Mixture F12 Ham's Liquid media (DMEM/F12, HyClone, GE Healthcare Life Sciences, Sweden) supplemented with 10% fetal calf serum (Sijiqing, Tianhang Biology, 3011-8611, China), Penicillin-Streptomycin Solution (Solarbio, P1400, China) (1:100 dilution) incubated at 37°C, 5% CO_2_ (Thermo Fisher Scientific, 3111, USA). The KGN cells were seeded on 96-well plates (5×10^3^cells/well). The cells were treated with various dosages of TG (1, 2, and 4 μM, Sigma-Aldrich, USA) for 48 hours and cells treated with PBS were used as controls. Cell viability was detected by 3-(4,5-dimethyl-2-thiazolyl) -2,5 -diphenyl-2-H -tetrazolium bromide (MTT) assays. According to MTT assay and ERS-mediated genes mRNA levels, we choose 1μM TG concentration for the subsequent experiments. KGN cell lines were cultured under normal condition, after replacing the entire medium with fresh medium, followed by incubation in DMEM/F12 with 1μM TG; PBS were used as control. The incubation time of the drug treatment was 48 hours.

### KGN cell culture and DHEA/PRE-084 supplementation

KGN cell lines were cultured under normal or TG-treated condition, after replacing entire medium with fresh medium, followed by incubation in DMEM/F12 with DHEA solution (10μmol/L, MedChem Express, China) [50, 91–93] or PRE084 solution (10μmol/L, MedChem Express, China) [94]; PBS were used as controls. The incubation time of the drug treatment was 48 hours.

### RNA extraction and qRT-PCR

Total RNA was extracted from GCs and KGN cells using Trizol (Ambin RNA, 15596-026, USA) and UNIQ-10 column total RNA extraction purification kit (Sangon biotech, SK1322, China). We used Thermo Scientific Reverted first strand cDNA synthesis Kit (Thermo, 00257208, USA) to synthesize cDNA. qRT-PCR assays were carried out by using ABI 7500 PCR Instrument (Applied Biosystems® 7500 Real-Time PCR, USA) in 20 μl reaction mixture containing 10 μl FastStart Universal SYBR Green Master (ROX) (Roche, Germany); 2.0 μl cDNA as template, with 0.2μl of each primer at 300nM and 7.6 μl of nuclease-free water. All primer synthesis was completed by Beijing DINGGUOCH ANGSHENG biotechnology company. Detailed information concerning primer sequences is shown in Supplementary Table 8. Results were quantified using Ct values (delta-delta Ct) method [95].

### DHEA supplementation in patients with DOR

It was a non-randomized concurrent control trial. The inclusion and exclusion criteria for patients with DOR were as previously described. 89 patients were divided into the DHEA supplementation group and control group according to patient preference. DHEA supplementation received a supplementation of DHEA 25 mg (GNC LiveWell, Pittsburgh, PA, US) three times per day [55, 86], more than 2 months in the ovarian stimulation process. No special treatment was given to the control patients. Because of several patients’ endometrial receptive were not suitable for embryo transfer, 41 women (14 women in the DHEA supplementation group and 27 women in the control group) underwent cleavage stage embryo transfers (ET) finally, each patient transferred less than 2 embryos.

### Statistical analysis

All cell experiments were performed in triplicate in different three times, so each quantitative data had 9 values. Statistical analysis of all data was performed using SPSS 19.0 statistical software (IBM Corp, USA). All values are given as group means ± SD, significance was set at p < 0.05.

### Ethical statement

All procedures were approved by Zhengzhou University ethics committee. All participating women gave signed informed consent.

## Supplementary Material

Supplementary Tables
