# Supplementary material for: Sigma-1 receptor is involved in diminished ovarian reserve possibly by influencing endoplasmic reticulum stress-mediated granulosa cells apoptosis
Source: Aging (Albany NY). 2020 May 14;12(10):9041–65. doi: 10.18632/aging.103166 (PMC7288944; doi:10.18632/aging.103166)
Supplement: Supplementary Tables [file aging-12-103166-s001..pdf]

SUPPLEMENTARY TABLES

Supplementary Table 1. Different clinical characteristics between women with DOR and NOR ($\bar{x} \pm s$).

	Normal ovarian reserve (NOR) (n = 84)	Diminished ovarian reserve (DOR) (n = 46)	*P*
Age of patients (year)	30.48±3.19	36.83±5.46	0.000**
Duration of infertility (year)	3.20±1.70	6.41±2.88	0.000**
Past NO. of IVF cycles	1.20±0.55	1.61±0.91	0.001**
Body mass index (BMI)	23.35±3.45	22.74±2.50	0.254
Antral follicular count (AFC)	12.21±4.73	4.22±1.66	0.000**
D3 FSH (IU/l)	5.54±1.38	10.38±4.69	0.000**
D3 LH (mIU/ml)	5.67±3.83	5.99±4.22	0.400
Serum AMH (ng/ml)	6.41±2.62	1.30±0.97	0.000**
Dosage of gonadotropin initiating (IU)	167.26±54.29	237.50±36.80	0.000**
Total dose of gonadotropin (IU)	1643.75±797.64	1932.07±987.21	0.073
Duraion of gonadotropin stimulation (days)	7.86±3.01	8.02±3.56	0.674
Endometrial thickness on ET day(mm)	10.45±1.75	10.17±1.81	0.346
No. of oocyte retrieved	11.89±6.23	4.11±2.83	0.000**
No. of available embryos	5.68±3.81	1.74±1.64	0.000**

**<0.01.

Supplementary Table 2. Low sigma-1 receptor protein in GCs of women with DOR ($\bar{x} \pm s$).

	Normal ovarian reserve (NOR) group (n = 3)	Diminished ovarian reserve (DOR) group (n = 3)	P
The level of sigma-1 receptor in GCs	183.01±15.07	107.81±21.94	0.006*

*<0.05.

Supplementary Table 3. Low sigma-1 receptor concentration in FF and serum of women with DOR ($\bar{x} \pm s$).

	Normal ovarian reserve (NOR) (n = 84)	Diminished ovarian reserve (DOR) (n = 46)	P
The level of serum sigma-1 receptor (pg/ml)	158.65±18.29	129.17±29.48	0.000**
The level of FF sigma-1 receptor (pg/ml)	164.76±16.38	130.90±18.88	0.000**

**<0.01.

Supplementary Table 4. High apoptosis index in GCs of women with DOR ($\bar{x} \pm s$).

	GCs of NOR group (n = 3)	GCs of DOR group (n = 3)	P
Early stage apoptotic rate (%)	10.42±1.46	21.21±1.75	0.001**
Late stage apoptotic rate (%)	17.95±0.35	22.44±2.32	0.030*
Total Early apoptotic rate (%)	28.37±1.16	43.65±2.00	0.000**

*<0.05; **<0.01.

Supplementary Table 5. The mRNA expression of apoptosis- and ERS-related genes in GCs of women with DOR ($\bar{x} \pm s$).

	GCs of NOR group (n = 3)	GCs of DOR group (n = 3)	P
Sigma-1 receptor	0.93±0.34	0.61±0.19	0.026*
BIP	0.87±0.33	1.29±0.46	0.041*
JNK	0.95±0.30	1.06±0.18	0.383
caspase-12	0.97±0.26	1.24±0.20	0.223
CHOP	1.18±0.34	1.72±0.49	0.038*
ATF4	1.03±0.27	2.19±0.09	0.002**
ATF6	1.17± 0.34	1.93 ± 0.51	0.002**
BCL-2	1.03±0.21	0.79±0.16	0.015*
BAX	0.99±0.16	1.40±0.12	0.034*
BCL-2/BAX(%)	96.28±13.16	64.12±12.53	0.000**

*<0.05, **<0.01.

Supplementary Table 6. Clinical outcomes in women with DOR with and without DHEA supplementation ($\bar{x} \pm s$).

	control women (n = 58)	DHEA administration women (n = 31)	P
Age of patients (year)	36.00±5.88	36.32±5.75	0.941
Duration of infertility (year)	6.12±2.90	7.23±2.90	0.106
Past NO. of IVF cycles	1.53±0.84	1.61±0.95	0.924
Primary/secondary infertility	24/34	16/15	0.355
Body mass index (BMI)	23.62±3.10	22.78±2.06	0.181
Antral follicular count (AFC)	3.81±1.65	4.13±1.94	0.593
D3 FSH (IU/l)	9.70±3.25	10.79±4.58	0.408
D3 LH (mIU/ml)	4.91±2.28	4.24±1.78	0.238
Serum AMH (ng/ml)	1.09±0.83	0.86±0.60	0.221
Total dose of Gn (IU)	1712.93±666.12	1683.06±688.19	0.843
Duraion of Gn stimulation (days)	7.76±3.33	6.97±2.94	0.653
Endometrial thickness on ET day(mm)	9.75±1.69	9.66±2.11	0.384
No. of oocyte retrieved	3.41±1.85	4.23±1.89	0.098
No. of available embryo	1.64±1.43	2.29±1.30	0.044*
High quality embryo rate (%)	50.52 (48/95)	45.07 (32/71)	0.486
No. of embryo transferred	1.57±0.50	1.69±0.608	0.573
Cycle cancelation rate (%)	36.21 (21/58)	16.13 (5/31)	0.047*
Clinical pregnancy rate (%)	29.63 (8/27)	35.71 (5/14)	0.691
Abortion rate (%)	25.00 (2/8)	20.00 (1/5)	1.000
Ectopic pregnant rate (%)	12.50 (1/8)	0.00 (0/5)	1.000

*<0.05.

Supplementary Table 7. Sigma-1 receptor protein levels in women with DOR with and without DHEA supplementation ($\bar{x} \pm s$).

	Control women (n = 3)	DHEA administration women (n = 3)	P
The level of sigma-1 receptor in GCs	98.37±15.86	135.03±16.85	0.052

Supplementary Table 8. Low apoptosis index in women with DOR with DHEA supplementation ($\bar{x} \pm s$).

	Control women (n = 3)	DHEA administration women (n = 3)	P
Early stage apoptotic rate (%)	21.34±1.76	17.50±1.31	0.002**
Late stage apoptotic rate (%)	23.91±1.61	21.41±1.04	0.088
Total Early apoptotic rate (%)	45.25±3.20	38.91±2.23	0.048*

*<0.05; **<0.01.

Supplementary Table 9. The mRNA expression in differently treated KGN cells ($\bar{x} \pm s$).

	No drug group (n = 3)	DHEA treated group (n = 3)	RPE-084 treated group (n = 3)	pcDNA3.1(+) group (n = 3)	pcDNA3.1(+) SIGMAR1 group (n = 3)	NC shRNA group (n = 3)	SIGMAR1 shRNA group (n = 3)	*P*
sigma-1 receptor	0.99±0.37	1.26±0.34	1.83±0.56	0.96±0.04	268.22±23.28	1.05±0.09	0.50±0.16	0.040[#]<br>0.000[△]<br>0.014[†]
BIP	1.06±0.45	0.89±0.35	0.85±0.64	1.02±0.24	0.89±0.16	0.99±0.28	2.53±0.74	0.016[†]
JNK	0.98±0.28	0.91±0.23	0.65±0.24	1.02±0.06	1.02±0.13	0.99±0.08	1.22±0.29	>0.05
caspase-12	1.02±0.08	0.76±0.19	0.89±0.44	0.99±0.02	0.97±0.06	1.06±0.37	1.65±0.52	>0.05
CHOP	1.00±0.24	0.71±0.23	0.51±0.05	1.00±0.10	0.77±0.15	1.00±0.28	1.44±0.28	0.045*<br>0.017[#]<br>0.048[△]<br>0.007[†]
ATF4	0.97±0.09	0.65±0.06	0.74±0.10	0.99±0.03	0.73±0.09	1.08±0.17	1.36±0.09	0.007*<br>0.045[#]<br>0.009[△]
ATF6	0.89±0.23	0.66±0.15	0.72±0.11	1.00±0.07	0.59±0.24	0.97±0.14	2.47±0.71	0.048[△]<br>0.022[†]
BCL-2	1.00±0.32	1.65±0.52	1.23±0.36	1.00±0.04	1.44±0.57	1.07±0.27	1.12±0.22	>0.05
BAX	1.00±0.11	0.73±0.29	0.79±0.24	1.00±0.07	0.81±0.24	0.99±0.02	1.86±0.22	0.017[†]
BCL-2/ BAX(%)	98.16±21.65	234.19±34.41	157.59±8.90	100.04±3.44	173.73±1.61	108.32±25.83	60.64±12.33	0.000*<br>0.012[#]<br>0.001[△]<br>0.003[※]

* No drug group VS. DHEA treated group, P<0.05;
[#] No drug group VS. RPE084 treated group, P<0.05;
[△] pcDNA3.1(+) group VS. pcDNA3.1(+) SIGMAR1 group,
[†] NC shRNA group VS. SIGMAR1 shRNA group, P<0.05.

Supplementary Table 10. MTT assay results for KGN cells treated with different concentrations of TG ($\bar{x} \pm s$).

TG(umol/L)	0 (n = 3)	0.25 (n = 3)	0.5 (n = 3)	1 (n = 3)	2 (n = 3)	4 (n = 3)	P
Cell viability (%)	100.00±1.59	97.00±1.44	95.68±0.83	77.17±2.06	72.90±1.96	70.75±3.47	0.072*<br>0.014[#]<br>0.000[△]<br>0.000[†]<br>0.000卍

* 0.25μmol/L TG group VS. control group;
[#] 0.5μmol/L TG group VS. control group;
[△] 1μmol/L TG group VS. control group;
[†] 2μmol/L TG group VS. control group;
卍 4μmol/L TG group VS. control group;

Supplementary Table 11. The ERS-related genes mRNA expression in TG- treated KGN cells ($\bar{x} \pm s$) (each group n = 3).

TG(umol/L)	0 (n = 3)	0.5 (n = 3)	1 (n = 3)	2 (n = 3)	4 (n = 3)	P
BIP	1.00±0.07	0.94±0.07	1.36±0.216	1.29±0.15	1.2459±0.26123	0.406*<br>0.049[#]<br>0.034[△]<br>0.187[†]
CHOP	1.00±0.05	0.96±0.06	1.26±0.05	1.30±0.16	1.28±0.16	0.490*<br>0.003[#]<br>0.038[△]<br>0.039[†]
BCL-2	1.02±0.05	0.98±0.08	0.82±0.04	0.78±0.06	0.75±0.02	0.534*<br>0.009[#]<br>0.008[△]<br>0.002[†]
BAX	0.99±0.05	0.91±0.06	1.18±0.19	0.99±0.15	0.96±0.11	0.135*<br>0.165[#]<br>0.926[△]<br>0.656[†]
BCL-2/BAX(%)	102.23±4.80	108.46±15.83	70.89±14.20	79.99±7.27	78.95±10.15	0.550*<br>0.022[#]<br>0.012[△]<br>0.023[†]

* 0.5μmol/L TG group VS. control group;
[#] 1μmol/L TG group VS. control group;
[△] 2μmol/L TG group VS. control group;
[†] 4μmol/L TG group VS. control group, P<0.05;

Supplementary Table 12. The apoptosis index in differently treated KGN cells ($\bar{x} \pm s$) (each group n = 3).

	Control gruop (n = 3)	TG-induced group (n = 3)	TG+PRE-084 group (n = 3)	TG +DHEA group (n = 3)	P
Early stage apoptotic rate (%)	6.21±1.17	27.84±2.60	14.13±1.37	16.45±2.75	0.000*<br>0.001#<br>0.006△
Late stage apoptotic rate (%)	13..23±1.19	17.34±3.19	15.76±1.04	15.62±1.44	>0.05
Total Early apoptotic rate (%)	19.44±1.80	45.18±5.60	29.89±2.01	32.07±4.09	0.002*<br>0.011#<br>0.031△

* TG-induced group VS.control group, P<0.05;
# TG-induced group VS.TG+PRE-084 group, P<0.05;
△ TG-induced group VS. TG+DHEA group, P<0.05.

Supplementary Table 13. Sigma-1 receptor protein levels in differently treated KGN cells ($\bar{x} \pm s$) (each group n = 3).

	Control group (n = 3)	TG-induced group (n = 3)	TG+PRE-084 group (n = 3)	TG +DHEA group (n = 3)	P
The level of sigma-1 receptor	403.74±72.19	317.31±55.55	577.82±130.95	449.91±52.78	0.034*<br>0.040#

* TG-induced group VS.TG+PRE-084 group, P<0.05;
# TG-induced group VS. TG+DHEA group, P<0.05.

Supplementary Table 14. The mRNA expression in KGN cells treated with different drugs ($\bar{x} \pm s$) (each group n = 3).

	Control group (n = 3)	TG-induced group (n = 3)	TG+PRE-084 group (n = 3)	TG +DHEA group (n = 3)	P
Sigma-1 receptor	1.26±0.27	0.96±0.21	1.50±0.14	1.30±0.12	0.021#
BIP	0.70±0.14	1.05±0.16	0.75±0.09	0.7623±0.07	0.049* 0.046# 0.046△
CHOP	0.71±0.14	0.99±0.04	0.76±0.10	0.76±0.11	0.029* 0.021# 0.025△
BCL-2	1.17±0.09	0.99±0.05	1.12±0.03	1.05±0.10	0.034* 0.016#
BAX	0.75±0.07	0.97±0.16	0.77±0.07	0.71±0.10	>0.05
BCL-2/ BAX(%)	157.28±25.42	103.72±20.77	147.20±15.13	148.70±14.51	0.048* 0.043# 0.037△
JNK	0.81±0.06	1.00±0.10	0.90±0.18	0.88±0.03	0.038*
caspase-12	0.86±0.04	1.01±0.08	0.83±0.11	0.86±0.07	0.039*
ATF4	0.88±0.03	0.98±0.09	0.77±0.07	0.73±0.12	0.012* 0.034# 0.041△
ATF6	0.74±0.05	0.96±0.12	0.71±0.05	0.85±0.05	0.045* 0.029#

* TG-induced group VS.control group, P<0.05;
# TG-induced group VS.TG+PRE-084 group, P<0.05;
△ TG-induced group VS. TG+DHEA group, P<0.05.
